# An Electronic Dashboard to Improve Dosing of Hydroxychloroquine Within the Veterans Health Care System: Time Series Analysis

**DOI:** 10.2196/44455

**Published:** 2023-05-12

**Authors:** Anna Montgomery, Gary Tarasovsky, Zara Izadi, Stephen Shiboski, Mary A Whooley, Jo Dana, Iziegbe Ehiorobo, Jennifer Barton, Lori Bennett, Lorinda Chung, Kimberly Reiter, Elizabeth Wahl, Meera Subash, Gabriela Schmajuk

**Affiliations:** 1 San Francisco VA Medical Center San Francisco, CA United States; 2 University of California San Francisco San Francisco, CA United States; 3 UCSF Philip R Lee Institute for Health Policy Studies San Francisco, CA United States; 4 VA Portland Health Care System Portland, OR United States; 5 Ralph H Johnson VA Medical Center Charleston, SC United States; 6 Palo Alto VA Medical Center Palo Alto, CA United States; 7 Stanford University Palo Alto, CA United States; 8 Raymond G Murphy VA Medical Center Albuquerque, AZ United States; 9 University of New Mexico School of Medicine Albuquerque, AZ United States; 10 Seattle/Puget Sound VA Healthcare System Seattle, WA United States; 11 UT Physicians Center for Autoimmunity Houston, TX United States

**Keywords:** medical informatics, patient safety, health IT, hydroxychloroquine, dashboard, Veterans Health Administration, audit and feedback, electronic health record

## Abstract

**Background:**

Hydroxychloroquine (HCQ) is commonly used for patients with autoimmune conditions. Long-term use of HCQ can cause retinal toxicity, but this risk can be reduced if high doses are avoided.

**Objective:**

We developed and piloted an electronic health record–based dashboard to improve the safe prescribing of HCQ within the Veterans Health Administration (VHA). We observed pilot facilities over a 1-year period to determine whether they were able to improve the proportion of patients receiving inappropriate doses of HCQ.

**Methods:**

Patients receiving HCQ were identified from the VHA corporate data warehouse. Using PowerBI (Microsoft Corp), we constructed a dashboard to display patient identifiers and the most recent HCQ dose and weight (flagged if ≥5.2 mg/kg/day). Six VHA pilot facilities were enlisted to test the dashboard and invited to participate in monthly webinars. We performed an interrupted time series analysis using synthetic controls to assess changes in the proportion of patients receiving HCQ ≥5.2 mg/kg/day between October 2020 and November 2021.

**Results:**

At the start of the study period, we identified 18,525 total users of HCQ nationwide at 128 facilities in the VHA, including 1365 patients at the 6 pilot facilities. Nationwide, at baseline, 19.8% (3671/18,525) of patients were receiving high doses of HCQ. We observed significant improvements in the proportion of HCQ prescribed at doses ≥5.2 mg/kg/day among pilot facilities after the dashboard was deployed (–0.06; 95% CI –0.08 to –0.04). The difference in the postintervention linear trend for pilot versus synthetic controls was also significant (–0.06; 95% CI –0.08 to –0.05).

**Conclusions:**

The use of an electronic health record–based dashboard reduced the proportion of patients receiving higher than recommended doses of HCQ and significantly improved performance at 6 VHA facilities. National roll-out of the dashboard will enable further improvements in the safe prescribing of HCQ.

## Introduction

Hydroxychloroquine (HCQ) is among the most commonly used medications for patients with autoimmune conditions and received special attention in 2020 as a potential treatment for COVID-19, resulting in drug shortages for chronic users [1]. These drug shortages, combined with recent guidelines emphasizing toxicities associated with long-term use, highlighted the issue of prescribing HCQ in appropriate doses. Long-term use of HCQ, especially at higher doses, can cause severe retinal toxicity in some patients. The risk of this toxicity is reduced if the average daily dose of HCQ is ≤5 mg/kg/day [2,3]. However, recent studies have revealed that 30%-40% of patients prescribed HCQ receive doses >5 mg/kg/day [4,5].

Previous studies have shown that enterprise-wide national dashboards are capable of improving care, but they have not been developed quickly enough, or disseminated widely enough, to make meaningful, population-level impacts on process or outcome measures [6-10]. Local, electronic health record (EHR)–based medication safety dashboards have been used to support medication safety but have not been scaled to date [11-14].

In this study, we sought to develop and deploy a national EHR-based medication safety dashboard within the Veterans Health Administration (VHA) to reduce inappropriate HCQ dosing. The VHA is the largest integrated health care delivery system in the United States, serving over 9 million veterans nationwide. Six VHA pilot facilities were enlisted to test the dashboard and invited to participate in monthly webinars. We followed pilot facilities over a 1-year period to determine whether they were able to improve the proportion of patients receiving inappropriate doses of HCQ.

## Methods

### Dashboard Development

The dashboard was developed as part of an ongoing project to improve the safe prescribing of high-risk disease-modifying antirheumatic drugs among VHA patients by the San Francisco VA’s Measurement Science Quality Enhancement Research Initiative. It was created using PowerBI (Microsoft Corp), a data management software package available within the VHA for approved users with secure access to EHR data. The VHA’s corporate data warehouse (CDW), which contains national VHA EHR data, served as the data source for the dashboard (Multimedia Appendix 1). PowerBI allows developers to extract, analyze, and display data from a variety of sources and features interactive tables and graphs that can be filtered or expanded using a graphical user interface [15]. Notably, PowerBI dashboards are “read-only,” that is, users can see and filter data elements, but in order to change data (eg, update the dose of HCQ), they must do so within the EHR.

### Facilities, Patients, and Data Elements

All 130 VHA facilities were eligible to be included in the study. We excluded 1 facility that had transitioned to the Cerner EHR and did not have patient data available in VistA, and 1 facility with fewer than 10 patients on HCQ, leaving 128 facilities for the analysis. Patients from included facilities in the VHA with a current, active prescription for HCQ were included in the data captured by the dashboard. Patients were excluded if the patient was deceased, or if the HCQ prescription indicated that it was a placebo or study drug. We extracted values for each patient’s most recently prescribed HCQ dose (in mg per day), derived from the “quantity dispensed” and “days-supply” fields in the medication order. We also extracted the most recently captured body weight (in kg) to calculate the HCQ dose in mg/kg/day. These data were then linked to Microsoft PowerBI Gateway servers, which are automatically updated every 24 hours to reflect new information from CDW (Multimedia Appendix 1).

### Dashboard Features

**Figure 1:**
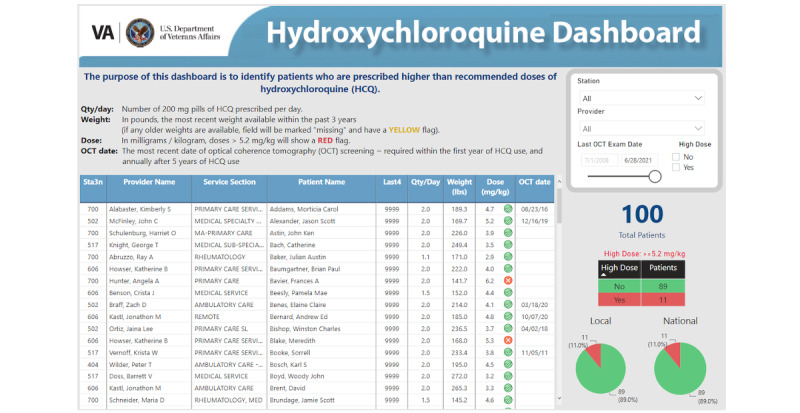
The hydroxychloroquine patient safety dashboard (using fictitious data). The dashboard was created using the Microsoft PowerBI software. Sta3n: unique medical facility codes for each VA station; Last 4: last 4 digits of a patient’s social security number needed to identify a patient in the computerized patient record system (CPRS).

Figure 1 illustrates the dashboard using fictitious patient data. The dashboard displayed patient identifiers (first and last name, last 4 digits of their social security number, and VHA facility), the number of HCQ pills prescribed per day, most recently documented weight, date of most recent documented optical coherence tomography (OCT) exam, and prescriber name and service. Calculated fields included HCQ dose in mg/kg/day based on weight and the number of pills prescribed per day. Rows were marked with a red x-mark if the HCQ daily dose was calculated to be ≥5.2 mg/kg/day (vs a green check mark if <5.2 mg/kg/day) in the column immediately to the right of the dose. Patients without a recorded weight within the past 3 years were flagged with a yellow circle, indicating missing data. Rows could be filtered by facility location, provider, OCT exam date, or by HCQ dose. The dashboard also displayed national-, facility-, and prescriber-level performance (proportion of patients with HCQ doses ≥5.2 mg/kg/day out of the total number of patients receiving HCQ) shown as pie charts for benchmarking. A user guide and video tutorial for the dashboard were available via a web-based link on the dashboard landing page. User interactions (number of times the dashboard is accessed per authorized user) were tracked using the PowerBI Activity Log feature [16].

### Study Period

Initial queries using CDW and PowerBI began in June 2020. The beginning of the study period—when baseline data collection on HCQ dosing across all VHA facilities started—began on August 11, 2020, prior to sharing the dashboard with any pilot testing facilities (see Multimedia Appendix 2). Of note, the final dashboard was developed over a period of under 5 months (June 2020 to October 2020).

### Pilot Testing Facilities

We enlisted rheumatology providers, pharmacists, and dermatologists from 6 VHA pilot facilities to test the dashboard between October 26, 2020, and December 6, 2021. Pilot facilities were selected based on their willingness to participate in a related study involving screening for infections prior to immunosuppression. The 6 VHA pilot facilities included Ralph H Johnson VA Medical Center, Charleston, SC; Palo Alto VA Health Care System, Palo Alto, CA; VA Portland Health Care System, Portland, OR; Raymond G Murphy VA Medical Center, Albuquerque, NM; San Francisco VA Medical Center, San Francisco, CA; and Puget Sound/Seattle VA Healthcare System, Puget Sound, WA.

Pilot facilities were invited to use the dashboard via email. Once they agreed, they were granted secure access along with any additional staff at that facility. All facilities were trained in the use of the dashboard via a web-based webinar. Site personnel were invited to participate in web-based meetings of a Rheumatology Quality and Safety Workgroup to share feedback, address any barriers, and update information on the use of the dashboard, every other month. Each facility leader was also sent a quarterly facility-specific report via email with run charts depicting the proportion of patients on HCQ at doses ≥5.2 mg/kg/day and the number of times their facility accessed the dashboard during that quarter.

Each pilot facility was encouraged to develop an individualized workflow for use of the dashboard. For example, some facilities would check the dashboard weekly or monthly, while others used the downloadable report feature to distribute flagged patients to individual providers or trainees. All facility workflows included review of the dashboard, review of EHR charts of flagged patients, and HCQ dose adjustment if appropriate.

### Control Facilities

Facilities in the control group did not have access to the dashboard and were not contacted as part of this study. Data on patients receiving HCQ were collected from CDW using the same process as was used for pilot facilities.

### Complex Medication Instructions and Policy Change

On the dashboard, HCQ dose in mg/kg/day was calculated based on the number of pills prescribed and the patient’s most recent weight. However, occasionally, HCQ orders had complex instructions (eg, “Take 2 pills Monday through Friday, and 1 pill on Saturdays and Sundays”), which resulted in miscalculations of the daily dose based on these fields. Two authors (AM and GS) reviewed 939 randomly selected charts and found 3% (28/939) of HCQ orders contained complex instructions. In order to reduce the chances of misclassifying patients as having an inappropriate HCQ dose due to complex instructions or fluctuating patient weights, on November 30, 2020, we made a policy change to designate doses of ≥5.2 mg/kg/day as “high dose” (as opposed to doses ≥5.0 mg/kg/day).

### Covariates and Descriptive Variables

We assessed facility characteristic variables that might be important in relation to medication safety practices in general and HCQ dosing specifically: facility region (Midwest, North Atlantic, Continental, Southeast, and Pacific); facility complexity (high, medium, and low); and the total number of patients prescribed HCQ at the facility [17].

In addition, we reported facility-level HCQ patient characteristics including the proportion of patients who were ≥55 years; self-identified non-Hispanic White, self-identified Hispanic or Latinx; with at least 1 VHA rheumatology clinic visit within 12 months of the beginning of the study period; and with a rural residence. Facility-level HCQ patient clinical factors included the proportion of patients with rheumatic diseases (rheumatoid arthritis, systemic lupus erythematosus [SLE], or other); with OCT exam documented; and the proportion with inappropriate HCQ dosing at baseline (August 11, 2020). A patient was considered to have a diagnosis if they had at least 2 codes (at least 30 days apart) for a specific condition listed here: rheumatoid arthritis, SLE, polymyalgia rheumatica, discoid lupus, nongout crystal arthropathy, undifferentiated connective tissue disease, sarcoidosis, antiphospholipid antibody syndrome, mixed connective tissue disease, systemic sclerosis, osteoarthritis, inflammatory myopathies (including polymyositis and dermatomyositis), psoriatic arthritis, ankylosing spondylitis, antineutrophil cytoplasmic antibody-associated vasculitis, other vasculitis (including Kawasaki disease), dermatitis, or giant cell arteritis.

### Statistical Analysis

Descriptive statistics were used to summarize facility characteristics and facility-level patient characteristics. We used interrupted time series (ITS) analysis to assess the effects of the dashboard on observed changes in the proportion of patients with HCQ doses ≥5.2 mg/kg/day. ITS is a strong quasiexperimental study design that can be used for single- and multiple group comparisons. In an ITS analysis, the outcome variable of interest (eg, the average proportion of patients with HCQ doses ≥5.2 mg/kg/day) is observed over multiple time periods before and after an intervention that is expected to “interrupt” the trend over time. ITS has been previously found useful when evaluating health care interventions for its ability to evaluate the causal impact of policy changes and health care interventions without random assignment [18,19]. We used the *itsa* command, which is available in the official Stata packages *newey* and *prais* [19].

Due to large variability in key facility characteristics observed at baseline between pilot and control facilities (proportion of patients on HCQ at doses ≥5.2 mg/kg/day, facility complexity, and mean number of patients prescribed HCQ at the facility; ITS regression output are displayed in Multimedia Appendix 3), we opted to implement a robust matching method using synthetic controls to measure the impact of the dashboard on HCQ dosing at the pilot facilities. Using this approach, pilot facility performance was compared to matched synthetic controls using the *synth* package in Stata [20]. Synthetic controls were constructed from a weighted combination of control units not exposed to the dashboard but with preintervention outcome dynamics and covariate levels similar to the pilot facilities prior to any interventions [21]. Matching was based on observed changes in the proportion of patients with HCQ doses ≥5.2 mg/kg/day, facility complexity, and the mean number of patients prescribed HCQ at the facility. To assess the balance of the pilot facilities and their synthetic controls, we used the absolute standardized mean difference (ASMD). As a rule of thumb, ASMD < 0.10 is an indicator of a good balance between synthetic control unit and a treated unit [22].

As part of the multiple group ITS analysis, pilot facilities were compared to synthetic controls in weekly increments of the proportion of patients with HCQ doses ≥5.2 mg/kg/day. We estimated the coefficients using segmented ordinary least square (OLS) linear regression models in which the errors were assumed to follow a first-order autoregressive process [19]. The model was specified to base the pooled autocorrelation estimate on the autocorrelation of the residuals. We expressed the effect of the dashboard on the proportion of patients with HCQ doses ≥5.2 mg/kg/day as intercept and slope changes. The intervention date was set as October 26, 2020 (the date the pilot facilities were granted access to the dashboard). We incorporated the policy shift (shift from recording the proportion of patients receiving ≥5.0 mg/kg/day to those receiving ≥5.2 mg/kg/day on November 30, 2020) using established methods [18].

Statistical analyses were performed using Stata 15 (StataCorp LLC). A *P* value <.05 was used as the criterion for statistical significance.

### Secondary Analyses

As secondary analyses, we compared the 6 pilot facilities to other facilities using modified Xbar-R charts. We used Microsoft QI Macros, a statistical process control software package plugin for Microsoft Excel, to generate modified Xbar-R charts to analyze the overall trends and stability in the proportion of patients with HCQ doses ≥5.2 mg/kg/day over time. Upper and lower control limits varied based on the average proportion of patients with HCQ doses ≥5.2 mg/kg/day. A continuous change of 6 or more points in a row or 8 or more points on the same side of the centerline is considered a significant trend [23].

We performed 2 separate comparisons: (1) pilot facilities versus all other facilities nationally and (2) pilot facilities versus matched control facilities. Matched control facilities were selected based on (1) the slope of proportion of patients prescribed HCQ at doses ≥5.2 mg/kg/day during the baseline period (August 11, 2020, to October 25, 2020); (2) the total number of patients prescribed HCQ; and (3) high facility complexity. Since pilot facilities had a mean) of 228 (SD 69) patients prescribed HCQ, we required matched control facilities to have at least 75 patients prescribed HCQ. Application of these criteria resulted in 8 matched control facilities, which were all included in the matched control sensitivity analysis.

### Feedback From Pilot Facilities

At the end of the study period, clinicians at pilot facilities were sent a confidential survey to solicit quantitative and qualitative feedback about the dashboard. The 14-item survey included questions about the capacity in which sites used the dashboard, usability of the dashboard, suggestions for improvement, and the likelihood of recommending the dashboard to a colleague or trainee.

### Ethics Approval

All VHA authors of this manuscript attest that the activities that resulted in producing this manuscript were conducted as part of a nonresearch evaluation under the authority of the National Rheumatology Field Advisory Committee and Center for Medication Safety. This work was approved by the VA Quality Enhancement Research Initiative (QUERI; IRB 15-18358).

## Results

### Pilot Facilities and Workflows

We identified 18,525 total users of HCQ nationwide in the VHA, including 1365 patients at the 6 pilot facilities. Across the 6 pilot facilities, 36 providers were granted access to the dashboard including 14 rheumatologists, 12 physician residents, 3 rheumatology fellows, 2 nurse practitioners specializing in rheumatology, 2 clinical pharmacists, 1 dermatologist, 1 registered nurse coordinator, and 1 primary care physician. Different pilot facilities developed different workflows around dashboard use to suit their needs. Some facilities requested access for all their clinicians (attendings and trainees) and had each one review their own patients. Others had a designated reviewer who checked the dashboard once a month or once a quarter. Another was able to download a spreadsheet containing the dashboard data and distribute it securely for clinician review (an example of typical dashboard clinic workflow for users is available in Multimedia Appendix 4). All pilot facilities had at least 20 interactions with the dashboard starting in October 2020; the median weekly number of dashboard interactions over the course of the study period was 8 (IQR 4-15).

### Baseline Facility Characteristics

Table 1 shows the characteristics of pilot facilities at all facilities nationally. Nationwide, at the start of the study period, 19.8% (3671/18,525; range 4.26% to 44%) of patients prescribed HCQ were receiving HCQ ≥5 mg/kg/day versus 16.1% (220/1365) among pilot facilities.

**Table 1:** Facility characteristics and practice-level patient characteristics for the pilot versus all facilities at baseline (November 8, 2020).

Facility characteristics			Pilot facilities (n=6)		All facilities (N=128)
**Complexity^a^, n (%)**					
	High complexity	6 (100)		84 (66)	
	Medium complexity	0 (0)		18 (14)	
	Low complexity	0 (0)		26 (20)	
**Geographic location, n (%)**					
	Continental	0 (0)		24 (19)	
	Midwest	0 (0)		26 (20)	
	North Atlantic	0 (0)		36 (28)	
	Pacific	5 (83)		22 (17)	
	Southeast	1 (17)		20 (16)	
Total patients prescribed HCQ^b^, mean (SD)			228 (69)		146 (107)
**Facility-level HCQ patient characteristics, mean (SD)**					
	Proportion of male patients	0.72 (0.05)		0.71 (0.10)	
	Proportion of patients aged >55 years	0.79 (0.10)		0.76 (0.09)	
	Proportion of non-White patients	0.33 (0.10)		0.32 (0.18)	
	Proportion of Hispanic/Latinx patients	0.05 (0.03)		0.05 (0.04)	
	Proportion of patients who visited a VA rheumatology clinic within 1 year of baseline	0.44 (0.08)		0.59 (0.19)	
	Proportion of patients with a rural residence	0.33 (0.02)		0.32 (0.21)	
**Facility-level HCQ patient clinical factors, mean (SD)**					
	Proportion of patients with rheumatoid arthritis^c^	0.43 (0.15)		0.45 (0.10)	
	Proportion of patients with systemic lupus erythematosus^c^	0.14 (0.02)		0.16 (0.04)	
	Proportion of patients with other rheumatic disease^c^	0.20 (0.12)		0.20 (0.12)	
	Proportion of patients with HCQ dose ≥5 mg/kg/day at baseline (August 11, 2020)	0.16 (0.03)		0.20 (0.07)	

^a^Station complexity: high complexity facilities have large levels of patient volume, patient risk, teaching and research, and contain level 4 to 5 intensive care units; medium complexity facilities have medium levels of patient volume, medium patient risk, some teaching and/or research, and contain level 3 and 4 intensive care units; low complexity facilities have the smallest level of patient volume, little or no teaching/research, the lowest number of physician specialists per patient, and contain level 1 and 2 intensive care units.

^b^HCQ: hydroxychloroquine.

^c^Rheumatic diseases were identified as veterans with 2 or more ICD-10 codes within the same disease category, separated by 30 or more days. Other autoimmune rheumatic diseases included: polymyalgia rheumatica, discoid lupus erythematosus, nongout crystal arthropathy, undifferentiated connective tissue disease, sarcoidosis, antiphospholipid syndrome, mixed connective tissue disease, systemic sclerosis, osteoarthritis, inflammatory myopathies (including polymyositis and dermatomyositis), psoriatic arthritis, ankylosing spondylitis, antineutrophil cytoplasmic antibody-associated vasculitis, and other vasculitis (including Kawasaki disease), lymphocytic infiltrates of the skin, or giant cell arteritis.

### ITS Analysis With Synthetic Controls

**Figure 2:**
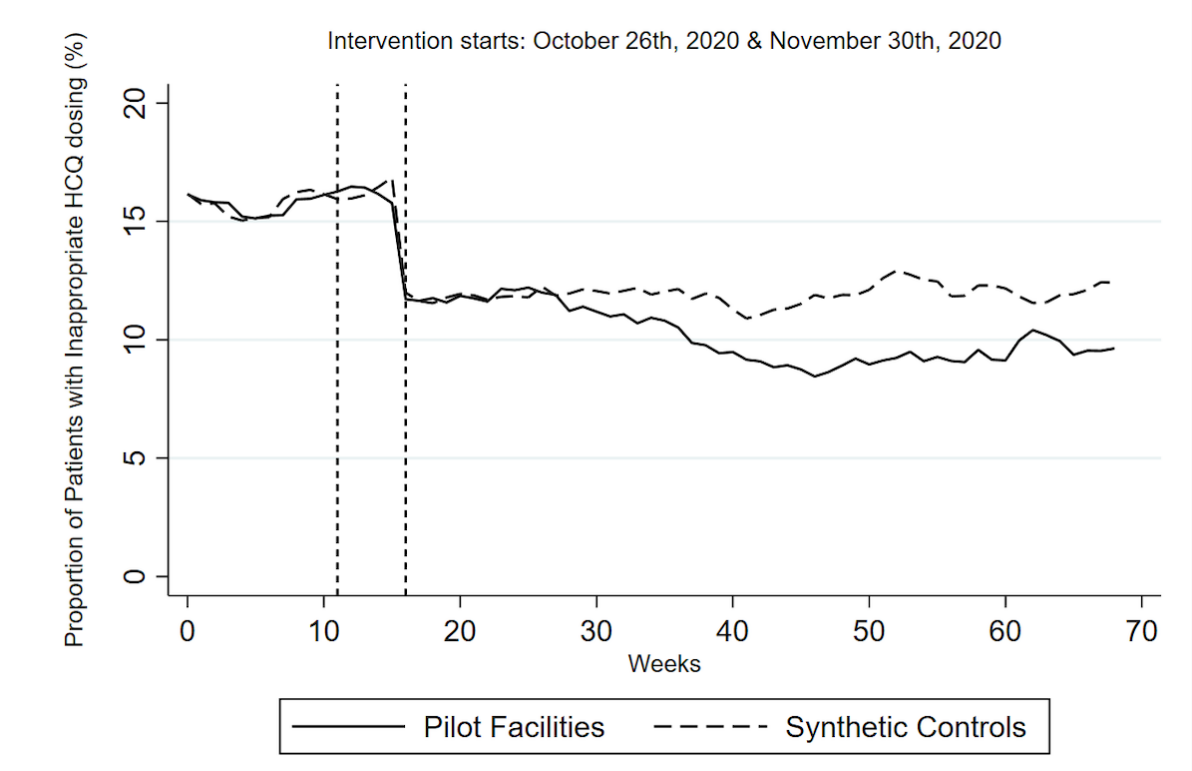
Synthetic control analysis with mean proportion of patients with high HCQ doses among the 6 pilot facilities and synthetic controls. The first intervention (October 26, 2020) was the date on which the dashboard was shared with the 6 pilot facilities. The second intervention (November 30, 2020) captured the policy change of adjusting the “high dose” definition ≥5.0 mg/kg/day to ≥5.2mg/kg/day to account for complex prescription instructions. HCQ: hydroxychloroquine.

Pilot facilities and synthetic controls were well matched in their predictor balance (ASMD=0.05). The postintervention linear trend showed pilot facilities’ proportion of patients with HCQ doses ≥5.2 mg/kg/day changed by –0.06 (95% CI –0.08 to –0.04) after the policy change, while the synthetic controls remained stable (0.006; 95% CI –0.00 to 0.01), with a statistically significant difference between the 2 groups by the end of the study period (–0.06; 95% CI –0.08 to –0.05; Multimedia Appendix 5 and Figure 2).

### Secondary Analyses

As seen in Multimedia Appendix 6, the modified Xbar-R control chart showed meaningful improvements in the proportion of patients receiving HCQ doses ≥5.2 mg/kg among pilot facilities over the course of the study period. There was a downward trend of 21 points outside of the upper and lower control limits, indicating a significant overall average process change. In contrast, the 8 matched control facilities’ proportion remained stable (ie, within the control limits). A comparison of pilot facilities to all other facilities nationally revealed similar results (Multimedia Appendix 7).

### Feedback From Pilot Sites

Six clinicians, 1 from each pilot facility, responded to the web-based survey. Of these 6 clinicians, 5 reported that the dashboard was extremely easy to use, 5 answered they were extremely likely to use the dashboard in the future, and 5 responded they were extremely likely to recommend the dashboard to a colleague or trainee.

## Discussion

In an era where the complexity of care and the number of evidence-based practices are ever expanding, the cognitive load required to address these practices during a short office visit can be overwhelming for clinicians. EHR-based dashboards are 1 method to support clinicians in evidence-based care of their patients. In this study, we developed an EHR-based medication safety dashboard to improve the safe prescribing of HCQ within the VHA. As part of a multipronged intervention, we found that audit and feedback via the dashboard resulted in a clinically meaningful and statistically significant reduction in the proportion of patients receiving high doses of HCQ among pilot facilities. Based on our linear postintervention trends, on the assumption that all facilities will behave similarly to the pilot sites, it would take approximately 4 years to reduce the proportion of patients receiving high HCQ doses from 16% to less than 5%.

Several features of the infrastructure available through the VHA made this a successful pilot. First, enterprise-wide PowerBI software was easily accessible as a pre-existing software suite available within the VHA for internal users. A new workspace was requested and granted within 48 hours; no new software installation was required. Second, it was straightforward to query VHA CDW data and then link these data to PowerBI servers. Construction of a first prototype of the dashboard took only a few months, and the final version (after several iterations) was available in 5 months. Beyond the VHA infrastructure, this pilot was feasible because of its limited scope to a single medication—HCQ comes in a single pill size, and most prescriptions have the number of pills dispensed corresponding to the daily dose, which facilitated calculating dose in mg/kg/day. Finally, because of its intuitive user interface, training required to use the dashboard by pilot sites was minimal.

There are few descriptions of EHR-integrated medication safety dashboards in the literature, and those that have been reported have also been successful [24]. For example, with the use of the UK SMASH dashboard, the prevalence of potentially unsafe prescribing of nonsteroidal anti-inflammatory drugs and other medications was reduced by 41% at intervention facilities [10,11]. Another, US-based, local, pharmacist-led medication safety program, which included a dashboard and educational outreach, reduced errors by 27%-49% after 6 and 12 months of use [25]. Several US patient registries have also developed clinician-facing dashboards to improve quality and medication safety and demonstrated significant improvements over time [26].

Although our pilot project was successful, we do note some limitations for the dashboard. First, although the development and validation of data in the dashboard were smooth, there were a small fraction of patients whose calculated doses remained inaccurate due to complex instructions that did not match the number of pills supplied. We attempted to mitigate these inaccuracies by only flagging doses ≥5.2 mg/kg/day instead of 5 mg/kg/day. We made this choice to avoid falsely labeling HCQ orders as high at the expense of missing some patients receiving doses above those recommended in the latest guidelines. Further work is needed to explore whether this tradeoff is worthwhile, especially since many clinicians use complex dosing in order to avoid average daily doses of ≥5 mg/kg/day, so the use of complex instructions may be correlated with appropriate dosing. In the future, 1 potential solution could be to develop an algorithm that captures information from the complex instructions using natural language processing techniques. Second, many pilot users requested additional features that are not available through PowerBI. Most importantly, users wished to be able to annotate dashboard tables directly or unflag patients who might be receiving higher than recommended doses of HCQ deliberately due to severe disease. Unfortunately, these features were not available in the VHA implementation of PowerBI at the time of this study.

One critical question for the future is whether the improvements observed in this pilot study will be sustainable. Clinician buy-in and ongoing utilization are crucial to the effectiveness of this dashboard as a sustainable audit-and-feedback tool [25]. Several of our pilot facilities started using this dashboard as a component of their routine quality improvement activities and reported dashboard use as part of a pay-for-performance program. Other facilities incorporated its use into trainee quality improvement activities. These additional use cases make sustainability more likely.

Another important question for future studies is about the clinical effects of reducing HCQ doses for some patients. Some recent observational studies have suggested that patients with SLE who decrease their HCQ dose may be at increased risk for disease flares [27,28]. It seems unlikely that small changes in dosing would have a large effect, but nevertheless, this is an important question to investigate. Unfortunately, since this is a national study limited by using structured EHR data, it is impossible to ascertain the condition of any specific patient before or after introducing the dashboard.

Moving forward, we will test the effects of the dashboard in a national roll-out across all VHA facilities. Additional mixed methods research will aid our understanding of provider adoption and sustained use of the dashboard and whether other interventions are needed to support safe prescribing of HCQ (eg, clinical decision support for weight-based dosing, or other pharmacy-based alerts or workflows) [29]. We also plan to roll-out additional dashboards focused on other important rheumatology safety issues, including pretreatment screening for latent infections in patients receiving biologic and targeted small molecule medications and HLA B:5801 testing for eligible patients receiving allopurinol. Our hope is that with a suite of dashboards and associated toolkits, quality improvement activities will be more feasible for all clinicians.

In summary, we successfully developed and deployed an EHR-based medication safety dashboard to improve the safe prescribing of HCQ within the VHA. The use of the dashboard significantly reduced the proportion of patients receiving higher than recommended doses of HCQ at 6 VHA facilities. National roll-out of the dashboard will enable further improvements in the safe prescribing of HCQ.

## Appendix

Multimedia Appendix 1 Architecture diagram of the hydroxychloroquine patient safety dashboard. The electronic health record data were pulled from the corporate data warehouse SQL Servers. The data were then directly linked from the SQL Servers to the PowerBI gateway and presented to the end user via a web interface.

Multimedia Appendix 2 Study timeline.

Multimedia Appendix 3

Multimedia Appendix 4 Hydroxychloroquine patient safety dashboard pilot facility Swimlane diagram.

Multimedia Appendix 5 Comparison of a linear postintervention trend for pilot versus synthetic control pilot facilities after the policy change (November 30, 2020).

Multimedia Appendix 6 Modified Xbar control chart showing mean percent of patients receiving inappropriate HCQ doses among pilot facilities and matched control facilities. The x-axis shows 2-week time segments during the study period from August 11, 2020, to December 6, 2021; the y-axis shows the percent of patients with higher than recommended HCQ doses. The vertical dotted lines denote the dates when the pilot facilities were granted access to the dashboard and when the “High Dose HCQ” definition changed from ≥5.0 mg/kg/day to ≥5.2 mg/kg/day. The orange and blue dotted lines show the average and upper/lower control limits for the 6 pilot facilities compared to 8 matched control facilities, respectively. The dots on the solid lines represent performance at each time point. CL: central line; LCL: lower control limit; HCQ: hydroxychloroquine; UCL: upper control limit.

Multimedia Appendix 7 Modified Xbar control chart showing mean percent of patients receiving inappropriate HCQ doses among pilot facilities and all other facilities nationally. The x-axis shows 2-week time segments during the study period from August 11, 2020, to December 6, 2021; the y-axis shows the percent of patients with higher than recommended HCQ doses. The vertical dotted lines denote the dates when the pilot facilities were granted access to the dashboard and when the “High Dose HCQ” definition changed from ≥5.0 mg/kg/day to ≥5.2 mg/kg/day. The orange and blue dotted lines show the average and upper/lower control limits for the 6 pilot facilities compared to all other 122 facilities, respectively. The dots on the solid lines represent performance at each time point. CL: central line; LCL: lower control limit; HCQ: hydroxychloroquine; UCL: upper control limit.

## Abbreviations

CDW: corporate data warehouse
EHR: electronic health record
HCQ: hydroxychloroquine
ITS: interrupted time series
OCT: optical coherence tomography
OLS: ordinary least square
SLE: systemic lupus erythematosus
VHA: Veterans Health Administration

## Data Availability

The data that support the findings of this study are available from the Department of Veterans Affairs, but restrictions apply to the availability of these data. Data are, however, available from the authors upon reasonable request and with permission of the Department of Veterans Affairs.
