# Supplementary material for: An Electronic Dashboard to Improve Dosing of Hydroxychloroquine Within the Veterans Health Care System: Time Series Analysis
Source: JMIR Med Inform. 2023 May 12;11:e44455. doi: 10.2196/44455 (PMC10221491; doi:10.2196/44455)

Multimedia Appendix 1. Architecture diagram of the hydroxychloroquine patient safety dashboard. The electronic health record data were pulled from the corporate data warehouse SQL Servers. The data were then directly linked from the SQL Servers to the PowerBI gateway and presented to the end user via a web interface.


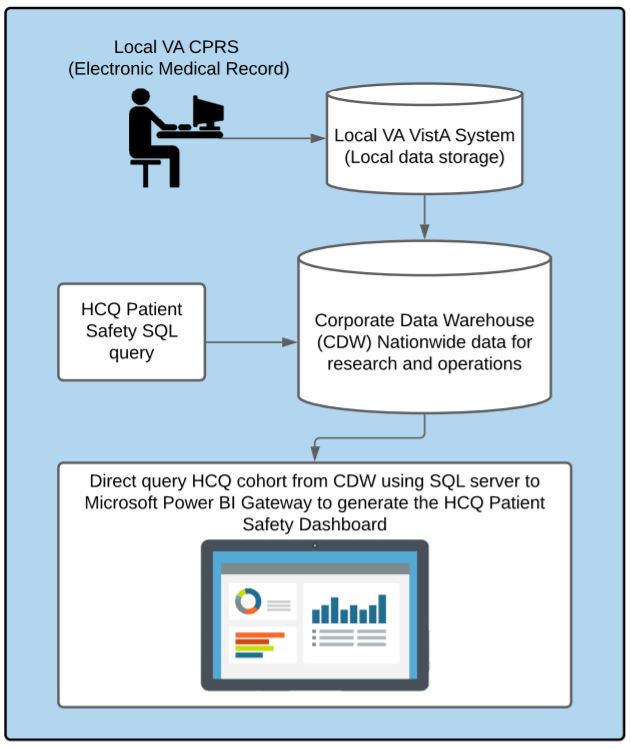

Supplement: Multimedia Appendix 1 [file medinform_v11i1e44455_app1.docx]
