# Supplementary material for: An Electronic Dashboard to Improve Dosing of Hydroxychloroquine Within the Veterans Health Care System: Time Series Analysis
Source: JMIR Med Inform. 2023 May 12;11:e44455. doi: 10.2196/44455 (PMC10221491; doi:10.2196/44455)
Supplement: Multimedia Appendix 2 [file medinform_v11i1e44455_app2.docx]

**Multimedia Appendix 2. Study timeline.**

| **Dates** | **Events** |
| --- | --- |
| June 15, 2020 | Initial queries using CDW and PowerBI |
| August 11, 2020 | Start of study period: first version of the hydroxychloroquine dashboard is complete, weekly data collection begins to establish baseline |
| October 26, 2020 | Dashboard shared with the six pilot facilities |
| November 23, 2020 | Initial feedback gathered from pilot site leaders |
| November 30, 2020 | Policy change - “High Dose” prescription of HCQ definition changed to ≥ 5.2 mg/kg/day |
| November 30, 2020 | Final design of dashboard complete |
| December 6, 2021 | End of study period |
