# Supplementary material for: An Electronic Dashboard to Improve Dosing of Hydroxychloroquine Within the Veterans Health Care System: Time Series Analysis
Source: JMIR Med Inform. 2023 May 12;11:e44455. doi: 10.2196/44455 (PMC10221491; doi:10.2196/44455)
Supplement: Multimedia Appendix 3 [file medinform_v11i1e44455_app3.docx]

Multimedia Appendix 3. Interrupted time series (ITS) analysis regression output with Newey-West SEs for pilot facilities versus all other facilities nationally (without synthetic control matching). As shown in the regression table, the initial mean level difference between pilot facilities and all other facilities nationally (_z) was significant (P<.001; 95% CI −5.95 to −3.90), but the difference in the mean baseline slope (_z_t) was not significant (P=.40; 95% CI −0.25 to 0.10). This is verified on inspection of the Xbar-R control chart (Multimedia Appendices 6 and 7): at baseline, the pilot facilities had a lower proportion of patients with high HCQ doses ≥5.2 mg/kg/day compared to other facilities. Given this mean level difference at baseline, we concluded that the pilot facilities were not comparable to other facilities nationally and that using these two groups in an ITS analysis could induce bias. Therefore, a more robust matching method based on the synthetic control approach was used in the primary analysis.

|  | **Coefficient** | **std. error** | **t** | **P-value** | **95% confidence interval** | |
| --- | --- | --- | --- | --- | --- | --- |
| _t | 0.071 | 0.079 | 0.89 | 0.372 | -0.085 | 0.226 |
| _z | -4.927 | 0.525 | -9.39 | <0.001 | -5.955 | -3.898 |
| _z_t | -0.075 | 0.089 | -0.85 | 0.397 | -0.250 | 0.099 |
| _x11 | -0.162 | 0.757 | -0.21 | 0.831 | -1.646 | 1.322 |
| _x_t11 | -0.051 | 0.256 | -0.2 | 0.841 | -0.553 | 0.450 |
| _z_x11 | 0.983 | 0.838 | 1.17 | 0.241 | -0.659 | 2.626 |
| _z_x_t11 | -0.072 | 0.262 | -0.28 | 0.782 | -0.587 | 0.442 |
| _x16 | -5.030 | 0.816 | -6.16 | <0.001 | -6.629 | -3.430 |
| _x_t16 | -0.023 | 0.236 | -0.1 | 0.923 | -0.486 | 0.440 |
| _z_x16 | 0.883 | 0.860 | 1.03 | 0.305 | -0.803 | 2.569 |
| _z_x_t16 | 0.093 | 0.242 | 0.39 | 0.700 | -0.381 | 0.567 |
| _cons | 20.631 | 0.474 | 43.49 | <0.001 | 19.701 | 21.561 |
