# Supplementary material for: An Electronic Dashboard to Improve Dosing of Hydroxychloroquine Within the Veterans Health Care System: Time Series Analysis
Source: JMIR Med Inform. 2023 May 12;11:e44455. doi: 10.2196/44455 (PMC10221491; doi:10.2196/44455)

Multimedia Appendix 4. Hydroxychloroquine patient safety dashboard pilot facility Swimlane diagram.


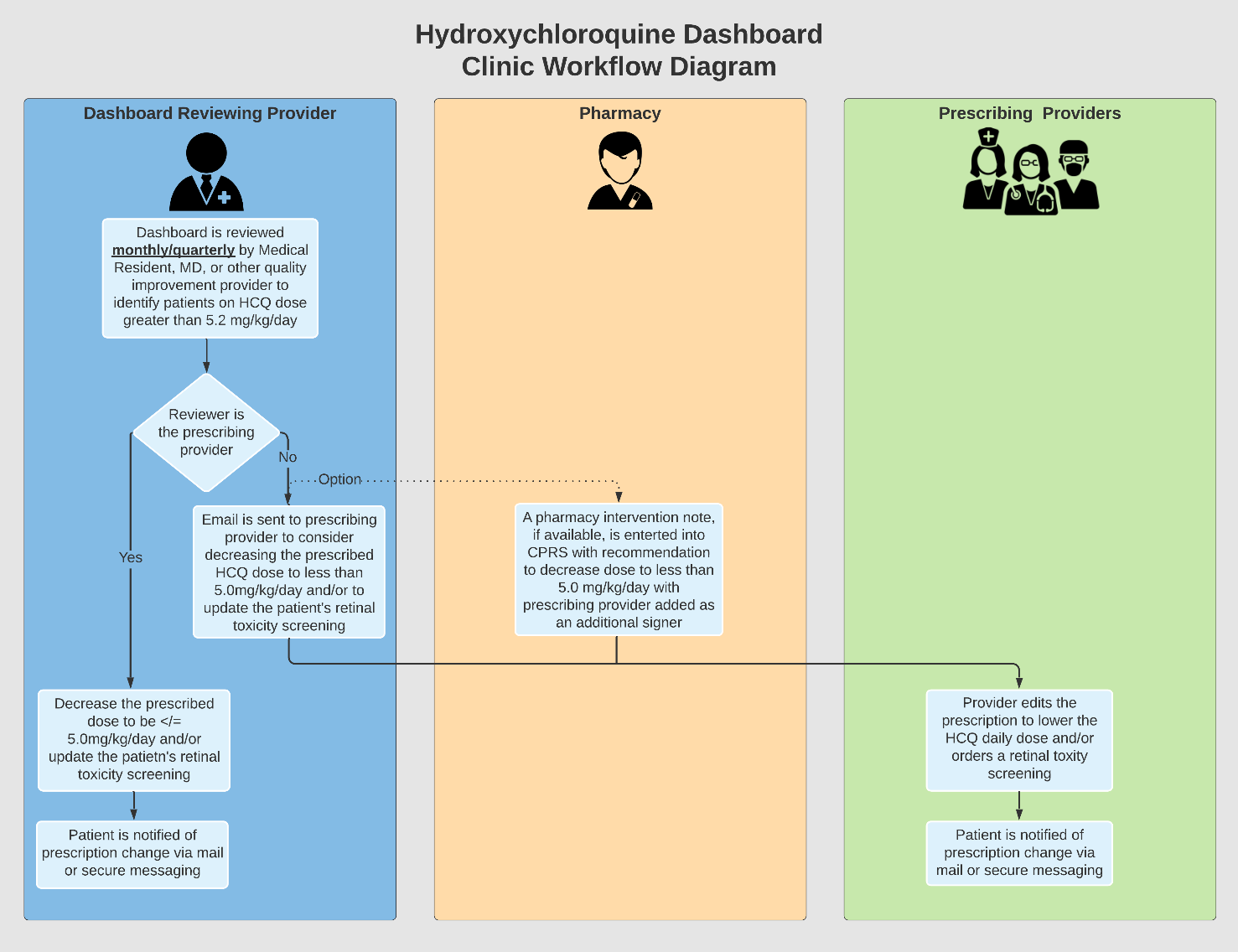

Supplement: Multimedia Appendix 4 [file medinform_v11i1e44455_app4.docx]
