# Supplementary material for: An Electronic Dashboard to Improve Dosing of Hydroxychloroquine Within the Veterans Health Care System: Time Series Analysis
Source: JMIR Med Inform. 2023 May 12;11:e44455. doi: 10.2196/44455 (PMC10221491; doi:10.2196/44455)
Supplement: Multimedia Appendix 5 [file medinform_v11i1e44455_app5.docx]

**Multimedia Appendix 5. Comparison of a linear postintervention trend for pilot versus synthetic control pilot facilities after the policy change (November 30, 2020).**

| **Linear Trend** | **Coefficient** | **Standard Error** | **T** | **p-value** | **95% Confidence Interval** |
| --- | --- | --- | --- | --- | --- |
| Pilot Facilities | -0.058 | 0.009 | -6.79 | <0.001 | (-0.075, -0.041) |
| Synthetic Control Facilities | 0.006 | 0.003 | 1.89 | 0.061 | (-0.000, 0.012) |
| Difference | -0.064 | 0.009 | -3.251 | <0.001 | (-0.082, -0.046) |
