# Supplementary material for: An Electronic Dashboard to Improve Dosing of Hydroxychloroquine Within the Veterans Health Care System: Time Series Analysis
Source: JMIR Med Inform. 2023 May 12;11:e44455. doi: 10.2196/44455 (PMC10221491; doi:10.2196/44455)
Supplement: Multimedia Appendix 6 [file medinform_v11i1e44455_app6.docx]

Multimedia Appendix 6. Modified Xbar control chart showing mean percent of patients receiving inappropriate HCQ doses among pilot facilities and matched control facilities. The x-axis shows 2-week time segments during the study period from August 11, 2020, to December 6, 2021; the y-axis shows the percent of patients with higher than recommended HCQ doses. The vertical dotted lines denote the dates when the pilot facilities were granted access to the dashboard and when the “High Dose HCQ” definition changed from ≥5.0 mg/kg/day to ≥5.2 mg/kg/day. The orange and blue dotted lines show the average and upper/lower control limits for the 6 pilot facilities compared to 8 matched control facilities, respectively. The dots on the solid lines represent performance at each time point. CL: central line; LCL: lower control limit; HCQ: hydroxychloroquine; UCL: upper control limit

Pilot Facilities (N= 6): Performance CL UCL/LCL

Control Facilities (N = 8): Performance CL UCL/LCL
